# A ketogenic diet rich in fish oil is superior to other fats in preventing NNK-induced lung cancer in A/J mice

**DOI:** 10.1038/s41598-024-55167-6

**Published:** 2024-03-07

**Authors:** Ingrid Elisia, Michelle Yeung, Sara Kowalski, Taras Shyp, Jason Tee, Serena Hollman, Amy Wong, Janette King, Roger Dyer, Poul H. Sorensen, Gerald Krystal

**Affiliations:** 1 The Terry Fox Laboratory, BC Cancer Research Centre, 675 West 10th Avenue, Vancouver, BC V5Z 1L3 Canada; 2 Department of Molecular Oncology, BC Cancer, Vancouver, BC V5Z 1L3 Canada; 3 Analytical Core for Metabolomics and Nutrition, BC Children’s Hospital Research Institute, Vancouver, BC Canada

**Keywords:** Ketogenic diet, Low carbohydrate, Lung cancer, Fish oil, Glycolysis, Cancer prevention, Lung cancer, Cancer metabolism, Cancer prevention, Lung cancer, Preclinical research, Cancer prevention, Lung cancer

## Abstract

Given that ketogenic diets (KDs) are extremely high in dietary fat, we compared different fats in KDs to determine which was the best for cancer prevention. Specifically, we compared a Western and a 15% carbohydrate diet to seven different KDs, containing either Western fats or fats enriched in medium chain fatty acids (MCTs), milk fat (MF), palm oil (PO), olive oil (OO), corn oil (CO) or fish oil (FO) for their ability to reduce nicotine-derived nitrosamine ketone (NNK)-induced lung cancer in mice. While all the KDs tested were more effective at reducing lung nodules than the Western or 15% carbohydrate diet, the FO-KD was most effective at reducing lung nodules. Correlating with this, mice on the FO-KD had low blood glucose and the highest β-hydroxybutyrate level, lowest liver fatty acid synthase/carnitine palmitoyl-1a ratio and a dramatic increase in fecal Akkermansia. We found no liver damage induced by the FO-KD, while the ratio of total cholesterol/HDL was unchanged on the different diets. We conclude that a FO-KD is superior to KDs enriched in other fats in reducing NNK-induced lung cancer, perhaps by being the most effective at skewing whole-body metabolism from a dependence on glucose to fats as an energy source.

## Introduction

Ketogenic diets (KDs) are high-fat, very low-carbohydrate (CHO) diets originally developed in the 1920s to treat intractable epilepsy^1^. Eating these diets typically results in 90% of total calories coming from fat, 8% from protein and only 2% from CHO^2^. Currently, there is a lot of excitement surrounding KDs, not only for weight loss but also for their potential as an adjuvant during cancer treatment^3–8^. This is because the switch from dependence on glucose to ketone bodies that are generated on a KD, is thought to hamper the growth of tumor cells, at least half of which are thought to be unable to use ketone bodies as an energy source^3–8^.

Given that KDs are extremely high in dietary fat, comprising anywhere from 70 to 90% of total calories, it is surprising that there has not as yet been a systematic evaluation of the fats typically consumed in a KD to prevent cancer. Related to this, it is highly likely that the type of fatty acid in a KD will have a significant impact on cancer cell proliferation and immune cell responses. For example, it has been shown that saturated fatty acids like palmitic acid, the most common dietary fatty acid, are potent activators of Toll like receptor 4 (TLR4) signaling in macrophages, making them pro-inflammatory^9^. As well, omega 6 fatty acids like arachidonic acid (AA)^10^ are known to be metabolized to prostaglandin E_2_ (PGE_2_), a prostanoid shown to help tumors grow, both directly and via suppression of anti-tumor immunity^11^. Omega 3 fatty acids, on the other hand, have been shown to be anti-inflammatory, at least in part by inhibiting AA conversion to PGE_2_^12^.

We previously established that reducing CHO levels from 50% of total calories, typically present in a Western diet, to 15%, and substituting easily digestible CHO (i.e., sucrose and amylopectin) with more resistant-to-digest CHOs (i.e., amylose or inulin) significantly lowers the number of NNK-induced lung nodules in A/J mice^13^. This low CHO diet (15%Amylose) contains 50% fat, with the fat composition being that typically consumed in a Western diet. Since KDs are becoming very popular and have even lower CHO levels (typically less than 5% of total calories) than our 15% Amylose diet, thus providing nascent cancer cells with less glucose, we asked if a KD would be superior to our low (15%)-CHO diet in preventing NNK-induced lung tumors in the A/J mice. As well, we asked if the type of fat consumed in a KD might influence its effect on NNK-induced lung nodules. Specifically, we compared the efficacy of a Western diet, our 15% amylose diet and seven KDs containing either Western type fats (the standard KD), or Western type fats enriched with medium-chain triglycerides (MCTs) (high in C8:0 caprylic acid), milk fat or butter (rich in the saturated fats myristic acid (C14:0) and palmitic acid (C16:0)), palm oil (high in the saturated fat, palmitic acid (C16:0)), olive oil (containing high levels of the omega 9 fatty acid, oleic acid (C18:1)), corn oil (high in the omega 6 fatty acid linoleic acid (C18:2)), or fish oil (high in the omega 3 fatty acids EPA (C20:5) and DHA (C22:6) (Suppl Tables 1, 2).

Because of its high fat content, there is also some concern that a KD may increase plasma levels of low-density lipoprotein (LDL)-cholesterol^14–16^. Herein, we have compared the effects of a Western diet with both a low CHO (15% amylose) diet and various KDs on both the incidence of NNK-induced lung nodules in mice, and on metabolic parameters, cytokine, chemokine and PGE_2_ levels, the levels of LDL-cholesterol and triglycerides, on liver damage, and the gut microbiome.

## Materials and methods

### Diets

All diets were formulated and custom-made in consultation with a dietitian (Jessica Flowers) at Envigo. The various KDs all contained 5% of total calories as amylose, 10% as casein and 85% as fats. The fats in these KDs were either Western type fats (standard KD) or Western type fats containing 30% of total calories as either medium chain fatty acids (MCT-KD) as a source of caprylic acid (C8:0), milk fat (MF-KD) rich in myristic (14:0) and palmitic (16:0) acids), palm oil (PO-KD) as a source of palmitic acid (16:0), olive oil (OO-KD) rich in oleic acid (18:1), corn oil (CO-KD) high in the omega 6 fatty acid, linoleic acid (18:2)) or fish oil (FO-KD) rich in the omega 3 fatty acids, eicosapentaenoic acid (EPA) and docosahexaenoic acid (DHA).

The different fats used were sourced by Envigo, except for the MCT oil (Bulletproof Brain Octane C8 MCT Oil) which we purchased from a local health food store. Corn oil, palm oil, olive oil, and lard used in the diets were from Columbus Vegetable Oils (Des Plaines, IL, USA). Fish oil was from OmegaPure, beef tallow from Chemol Company (Greensboro, NC, USA) and cocoa butter was from Arista Industries (Wilton, CT, USA). Briefly, the fat sources were first whipped, followed by the addition of casein, melted cocoa butter, fiber, starches, vitamins, and minerals. The diets were vacuum-sealed in 1 kg packs, double bagged, and irradiated before shipment to our facility. Upon arrival, all diets were frozen at – 20 °C, and thawed at 4 °C 1 week before use.

### Animals

**Figure 1:**
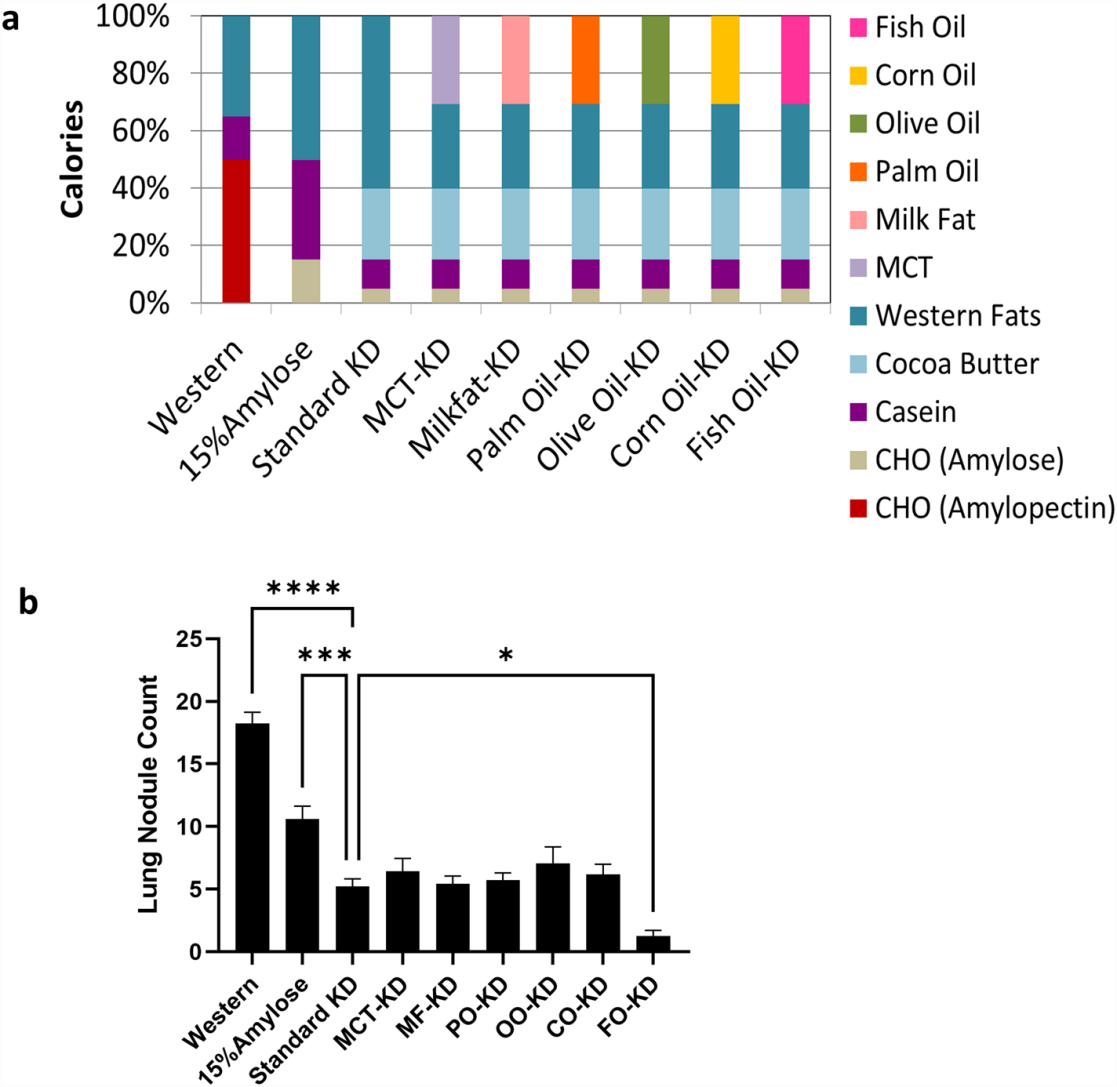
A ketogenic diet enriched in fish oil is most effective at lowering NNK-induced lung nodule formation in A/J mice. (**a**) Composition of the various isocaloric diets used in this study. (**b**) Female A/J mice (n = 12–14) were put on the diets shown in (**a**) at 12 weeks of age and then NNK injected twice, 1 week apart, starting 2 weeks later and remained on their respective diets ad libitum and euthanized after 20 weeks. Surface tumor nodules were counted by two blind counters. *Denotes significance (P < 0.05) compared to mice fed with the standard KD.

Female A/J mice (4–6 weeks old) were purchased from the Jackson Laboratories (Bar Harbor, ME, USA). Mice were acclimatized in our pathogen-free facility at the BC Cancer Research Centre and fed a standard rodent chow (Envigo #2920) until they reached 12 weeks of age. During this time the mice were housed in double-decker cages, composed of two stacked rat cages with a hole cut between them to allow mice to move between levels. These double-decker cages had one exercise wheel on each level to promote exercise. The mice were then kept in the double-decker cages but randomly switched to one of the isocaloric diets (n = 12–14 per diet) described in Fig. 1a, Suppl Tables 1, 2 for 2 weeks, before being injected twice with 50 mg/kg nicotine-derived nitrosamine ketone (NNK), 1 week apart. To maintain the KDs in a semi-solid state, we incorporated a fixed amount of cocoa butter to all the KDs. The mice then remained on their respective diets ad libitum and were euthanized after 20 weeks. Mice fed with the Western diet served as a positive control. All animal experiments were reviewed and approved by the University of British Columbia Animal Care Committee (A22-0072). All experiments were performed in accordance with relevant guidelines and regulations. The study was carried out in compliance with the ARRIVE guidelines (https://arriveguidelines.org). Mice that required anti-inflammatory drugs or antibiotics during the experiment, due to fight wounds or unrelated events, were excluded from the study.

Mice were euthanized between 8 to 10 a.m. by anesthesia with isoflurane followed by CO_2_ asphyxiation and cervical dislocation. Mice were then cardiac-punctured, and the blood collected into EDTA-coated tubes, which were then centrifuged at 6000*g* for 5 min at 4 °C. The plasma was collected and stored at − 80 °C for biochemical analysis. The lungs were collected and rinsed once in PBS and surface tumor nodules counted by two blind counters using a Leica MZ9.5 microscope (Meyer Instruments, Houston, TX). The lungs were then blotted dry and frozen at − 80 °C.

Lungs and livers were fixed in formalin for 48 h, washed with PBS twice, and the lung surface tumors counted before putting the lungs into 70% ethanol for storage at 4 °C. The lungs were paraffin-embedded, sectioned at 4 µm, and stained with H&E by the histotechnologists at the Centre for Translational and Applied Genomics (CTAG).

### Blood glucose

Mouse blood glucose levels were measured between 10 p.m. and 2 a.m., when they were actively feeding, using a One Touch Verio Reflect^®^ blood glucose monitor and blood collected from the tail vein.

### Biochemical analysis

Plasma β-hydroxybutyrate (β-HB, Cayman Chemical, Ann Arbor, MI), alanine aminotransferase, and cholesterol (Abcam, Cambridge, MA) levels were quantified using kits according to the manufacturer’s instructions. The expression of pro-inflammatory cytokines and chemokines in mouse lungs, including IFN-γ, IL-1β, IL-2, IL-4, IL-5, IL-6, IL-10, IL-12p70, KC/GRO, TNF-α, as well as PGE_2_ was quantified using the V-PLEX Proinflammatory Panel 1 Mouse Kit (Meso Scale Discovery, Rockville, MD) and an ELISA kit from Cayman Chemical (Ann Arbor, MI), respectively, according to the manufacturer’s instructions. The preparation of lungs for these analyses was adapted from Elisia et al.^17^. For Mesoscale analysis, 100 µg of protein, as determined by BCA assays, was loaded per well.

### Immunohistochemistry

Paraffin-embedded, sectioned lung tissue was stained by immunohistochemistry for Ki67 (Abcam, ab16667 1:100), pERK (Cell Signaling Technology, CST4376S, 1:400), fatty acid synthase (FAS) (Cell Signaling Technology, CST 3180, 1:100), and COX2 (Cell Signaling Technology, CST12282S 1:1000) as previously described^17^. For each mouse, 3–10 pictures were taken at 200× power using a Leica DM2500 camera (Meyer Instruments, Houston, TX). TUNEL assays were performed using an In Situ Cell Death Kit, POD (Sigma-Aldrich #11684817910) according to the manufacturer’s instructions^18^. Ki67-positive and TUNEL-positive cells were expressed as a percent of the total cells counted within the field using ImageJ software. The pERK, FAS, and COX2 stained slides were evaluated based on their immunoreactive score (IRS), which is a product of their positivity score (attributed 1–4 according to the percent of positive cells), and the intensity score (0–3), as previously described^19^. Each slide was scored by two independent reviewers, and the average score is reported.

### Liver TBARS determination

Determination of the level of thiobarbituric acid substances (TBARS) within the livers of the mice on the different diets was adapted from the Cayman Chemical TBARS (TCA assay) protocol. Briefly, frozen liver (100 mg/mL) was dounce homogenized in RIPA buffer supplemented with a cocktail of protease inhibitors (phenylmethylsulfonyl fluoride, aprotinin, and leupeptin). After rotation for 30 min at 4 °C, the liver lysate was centrifuged at 16,060*g* for 10 min at 4 °C. The supernatant was collected and used to determine protein content by the BCA assay. To measure TBARS, 100 µL of sample or standard (1,1,3,3-Tetramethoxypropane) was mixed with an equal volume of 10% TCA, and 800 µL of thiobarbituric acid solution (106 mg of thiobarbituric acid made in 10 mL of 20% acetic acid and 10 mL 0.7 N NaOH). The mixture was then boiled for 1 h, after which the tubes were centrifuged for 10 min at 1600*g* at 4 °C. The supernatant (200 µL) was plated in a flat bottom 96 well plate and read at 540 nm.

### Liver 8-OHdG determination

Liver 8-OHdG levels were determined using an ELISA kit from Abcam (#ab201734) according to the manufacturer’s instructions. Briefly, liver DNA from homogenized lysates was extracted using DNeasy Blood & Tissue Kits (Qiagen, Valencia, CA). DNA was heated at 100 °C for 10 min and digested using nuclease P1 (Sigma-Aldrich, N8630), prepared in 20 mM sodium acetate buffer (pH 5.3), 5 mM ZnCl_2_, and 50 mM NaCl for 1 h at 37 °C. One unit of alkaline phosphatase was added per 100 µg DNA and the mixture was incubated at 37 °C for 1 h before deactivation by heating with 1/10 volume of 200 mM EGTA for 10 min.

### Western blotting

The FAS and carnitine palmitoyltransferase 1A (CPT1a) expression levels in the livers were determined by Western blots as previously described (16) using anti-FAS antibodies from Cell Signaling Technology (1:1000, #3180) and anti-CPT1a antibodies from Abcam (1:1000, #ab234111). GAPDH was used as a loading control.

### Determination of fatty acid profile in mouse lungs

The fatty acid composition of the mouse lungs was performed by the Analytical Core for Metabolomics and Nutrition at the BC Children’s Hospital Research Institute, as previously described^17^.

### Feces collection

A week before the mice were euthanized, feces were collected with autoclaved forceps from mice singly placed in an empty, sterile Allentown cage. We collected four fecal pellets per mouse in two separate microfuge tubes and placed the tubes immediately on dry ice. The feces were stored at − 80 °C until analysis. The feces were processed for 16S sequencing by Microbiome Insights (Vancouver, BC).

### Assessment of liver damage

To assess liver damage, whole liver sections stained with hematoxylin and eosin (H&E) were analyzed blindly by a certified pathologist. Scoring was based on four major histological criteria for non-alcoholic fatty liver disease (NAFLD) and was analyzed semi-quantitatively^20^. Briefly, steatosis was scored from 0 to 4 based on the percentage of liver parenchyma affected by lipid degeneration: 0 =  < 5% of hepatocytes with lipid vesicles; 1 = 5–33%; 2 = 34–66%; 3 =  > 66%. Scoring of the ballooning degeneration, a major indicator of hepatocyte degeneration during acute liver injury^21^, was performed in the following way: 0 = no ballooning hepatocyte injury, 1 = few distinctive ballooned hepatocytes; 2 = multiple foci or prominent ballooning degeneration of hepatocytes. Liver inflammation was scored based on the number of inflammatory foci per 20× field of view: 0 = none, 1 =  < 2 foci/20× field of view (fov); 2 = 2–4 foci/20× fov; 3 =  > 4 foci/20× fov. The presence and degree of fibrosis were assessed using H&E and supported with Mallory's Trichrome staining (Abcam #150686): 0 = none; 1 = perisinusoidal; 2 = perisinusoidal and portal/periportal, 3 = bridging fibrosis, 4 = cirrhosis.

The cumulative score for the staging of NAFLD in the experimental groups was based on the histological activity of NAFLD (i.e., steatosis, ballooning degeneration, and inflammation), and the degree of fibrosis. The stage of NAFLD was calculated based on the staging system previously described^22^ as well as the degree of fibrosis to give the total score number: score 1–4 = mild degree of NAFLD; score 5–8 = moderate NAFLD; score 9–12 = severe form of NAFLD.

### Statistics

All statistics were performed using GraphPad Prism 9.5.1. The effect of the diets relative to the standard KD, which contains fats typically found in a Western diet, on all end-point measures was evaluated using one-way ANOVA, using Dunnette posthoc analysis to correct for multiple comparison tests. For fatty acid composition, Tukey’s posthoc analysis comparing the level of individual fatty acids from all diets was performed. P < 0.05 was considered statistically significant. For fecal microbiome analysis, alpha diversity was estimated with the Shannon index on the raw operational taxonomic unit (OTU) abundance tables after filtering out contaminants. The significance of diversity differences was performed using ANOVA. For beta diversity analysis, OTUs occurring with a count of less than 3 in at least 5% of the samples were removed and Bray–Curtis indices were computed. Variation in community composition was assessed with permutational multivariate analysis of variance (PERMANOVA) with the treatment group as the main fixed factor and using 999 permutations for significance testing. Pairwise contrasts between groups were calculated, and the p-values were corrected for multiple comparisons using the FDR method. For differential abundance testing, *Linda* function from MicrobiomeStat package was used to identify differentially abundant taxa between the standard KD and the FO-KD diets. All fecal microbiome analysis was performed in the R environment.

## Results

### A ketogenic diet containing fish oil is most effective at reducing NNK-induced lung cancer

To compare the effects of a Western diet with both a low CHO (15% amylose) and various KDs on the incidence of NNK-induced lung nodules in mice, we fed female A/J mice the isocaloric diets shown in Fig. 1a and Suppl Tables 1, 2, for 2 weeks prior to NNK injections and then kept them on these diets until the mice were euthanized 5 months later. The Western diet reflected a typical human Western diet^23^ and contained 50% of total calories as easily digestible CHO (sucrose and amylopectin), 15% as casein and 30% as typical Western diet fats. The low CHO 15% amylose diet contained 15% of total calories as resistant starch (amylose), 35% as casein and 50% as Western fats. The various KDs all contained 5% of total calories as amylose, 10% as casein and 85% as fats. These fats were either Western type fats (standard KD) or Western type fats containing 30% of total calories as either medium chain fatty acids (MCT-KD), milk fat (MF-KD), palm oil (PO-KD), olive oil (OO-KD), corn oil (CO-KD) or FO (FO-KD).

As shown in Fig. 1b, mice (n = 12–14) fed the Western diet had, on average, 18 lung nodules/mouse, while mice on the 15% amylose diet had approximately 10 nodules/mouse, in keeping with our previous results^13^. The standard KD significantly reduced lung nodules, compared to both the Western and 15% amylose diet (P < 0.05). Since a KD is made up of a large proportion of fat, we then asked if the type of fat incorporated into a KD might influence lung tumor formation. Interestingly, we observed that substituting the fats in a standard KD, containing typical Western diet fats, with those specifically enriched in MCT, milk fat/butter, palm oil, olive oil, or corn oil had no further impact on lung nodule formation, suggesting these types of fats were neither beneficial nor detrimental to the pathogenesis of lung cancer in this model. However, when FO was incorporated into the KD, we saw a further significant (P < 0.05) reduction in lung nodule numbers. Thus, a FO-KD appears to be superior to other fat-containing KDs in preventing NNK-induced lung cancer formation.

### Incorporation of fish oil into a ketogenic diet increases ketone bodies more than other fats

**Figure 2:**
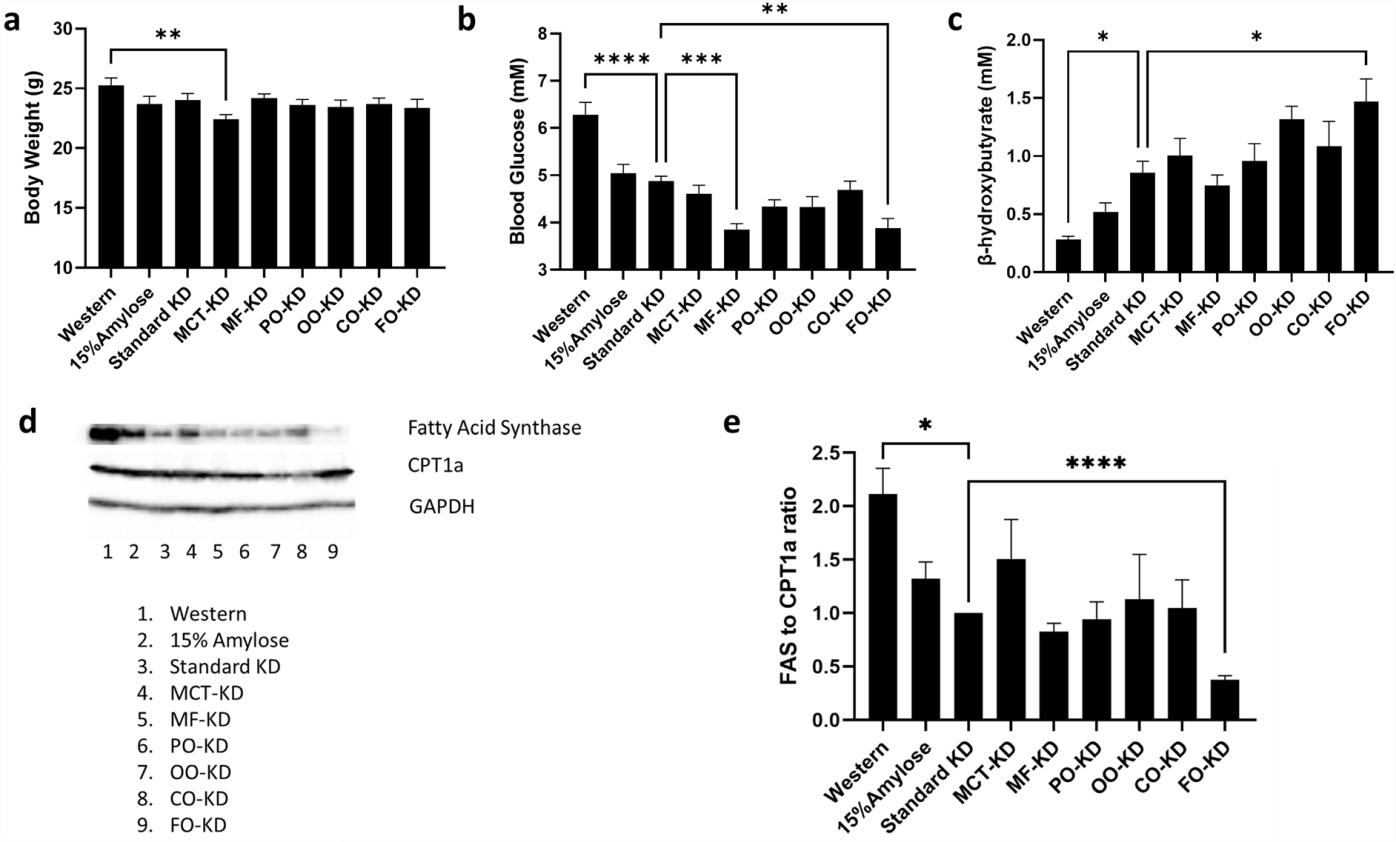
Effect of the diets on (**a**) body weight, (**b**) blood glucose, (**c**) plasma β-HB, (**d**) liver fatty acid synthase and CPT1a levels. Representative uncropped blots are shown in Suppl Fig. 5) and (**e**) FAS to CPT1a ratio (n = 11–14). *Denotes significance (P < 0.05) compared to mice fed with the standard ketogenic diet.

To gain some insight into the mechanism(s) responsible for the superiority of the FO-KD at reducing NNK-induced lung nodules, we first compared the body weights of all the mice just prior to euthanasia. As shown in Fig. 2a, there was no significant difference between the groups on their isocaloric diets, other than the mice on the MCT-KD were slightly lighter. We next asked whether the decrease in tumor nodules in KD-fed mice, and specifically in FO-KD-fed mice, correlated with any changes in their metabolisms. As expected, mice fed a KD had significantly (P < 0.05) lower post-prandial blood glucose levels than mice fed a Western diet (Fig. 2b). Moreover, mice fed KDs enriched in milk fat or FO had blood glucose levels that were significantly (P < 0.05) lower than the other KDs (Fig. 2b). In keeping with the low blood glucose levels, mice on the KDs had significantly higher levels of the plasma ketone body, beta-hydroxybutyrate (β-HB) than mice on the Western diet and were at levels suggesting the diet promoted ketogenesis in these mice (Fig. 2c). As can be seen comparing Fig. 2b and c, there was an inverse relationship between β-HB levels and blood glucose levels, as might be expected. Of the fat types tested, only the FO-KD had β-HB levels that were significantly (P < 0.05) increased over that in mice fed a standard KD, which corresponded with the unique ability of the FO-KD to further lower lung nodule formation in these A/J mice. This is consistent with our observation that the FO-KD yielded the lowest expression of FAS and the highest level of CPT1a within the liver, as assessed by Western blots (Fig. 2d). Expressed as the FAS/CPT1a ratio, FO-KD was the only KD that further significantly (P < 0.05) lowered the FAS/CPT1a ratio relative to mice fed a standard KD (Fig. 2e). A higher level of ketosis may thus be responsible, at least in part, for the NNK-induced lung nodule-lowering properties of the FO-KD.

### The fatty acid composition within the lungs of the mice reflects the levels in their diets

**Figure 3:**
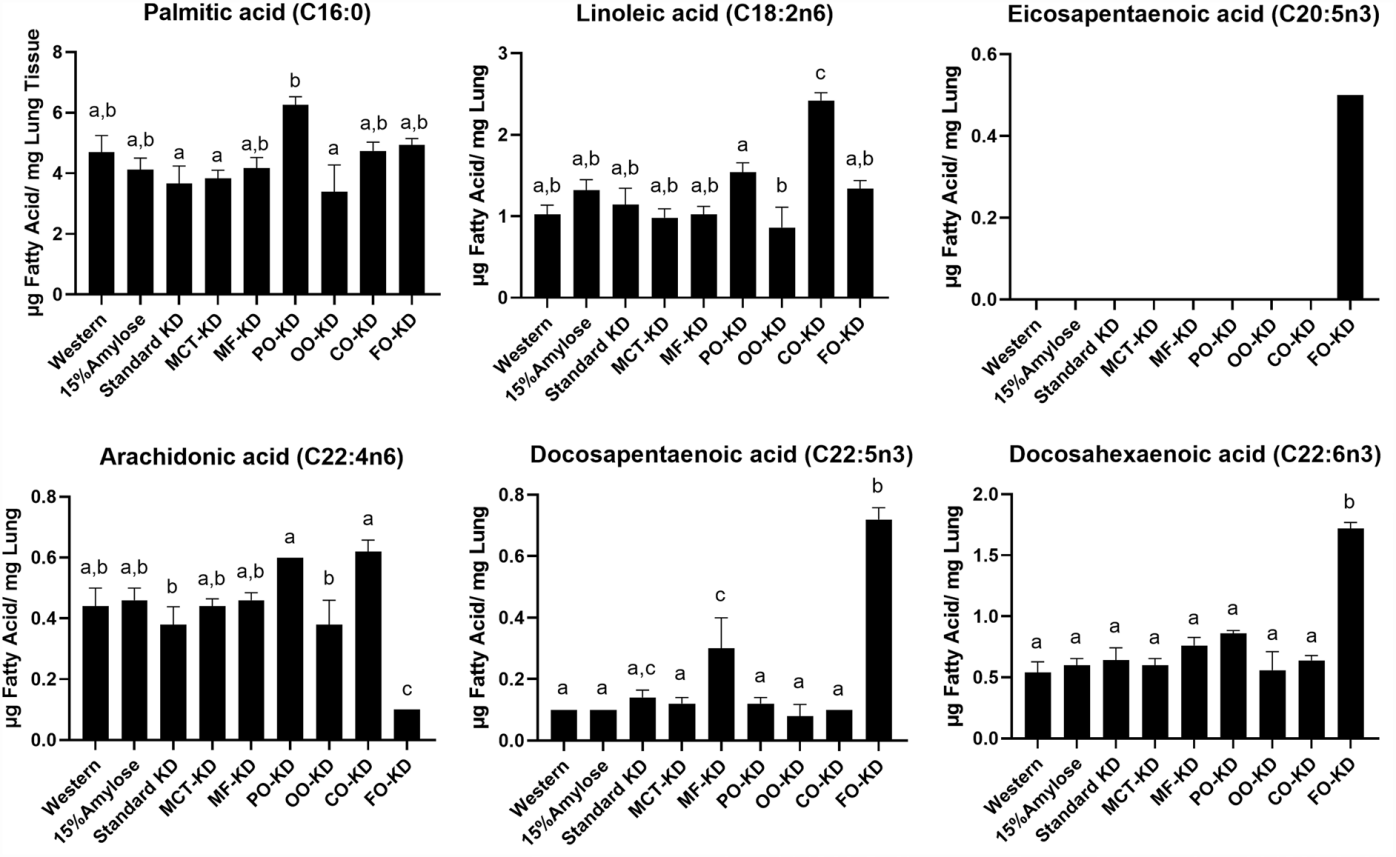
The fish oil containing ketogenic diet uniquely increases EPA, DPA and DHA while decreasing AA in the lungs. n = 5 and the diets not sharing the same letter denote a significant (P < 0.05) difference.

To determine if the reduction in lung nodules was associated with changes in specific fatty acids in the lungs, we performed a fatty acid analysis of the lungs (Fig. 3). Of all the KDs, CO-fed mice had significantly (P < 0.05) increased linoleic acid when compared to mice fed the standard KD. Also of note, PO-fed mice had one of the highest palmitic acid levels in their lungs, as might be expected. As can also be seen in Fig. 3, incorporating FO into the KD significantly (P < 0.05) reduced the omega 6 fatty acid, AA, while at the same time increasing EPA, DPA, and DHA omega-3 fatty acids. The highest levels of myristic acid were found, interestingly, in both the FO-KD and the Western diets. As a percent of total fatty acids, oleic acid was the dominant fatty acid found in OO-KD fed mice (Suppl Fig. 1). Since the inclusion of MCT oil, palm oil, corn oil, olive oil, and milk fat into the KD had no impact on lung nodule number, while the incorporation of FO further reduced lung tumor formation, it is reasonable to suggest that the increase in EPA, DPA, and DHA within the lungs were more important than other fatty acids in influencing the number of lung cancer nodules.

### The fish oil-containing ketogenic diet reduces PGE_2_ in mouse lungs

**Figure 4:**
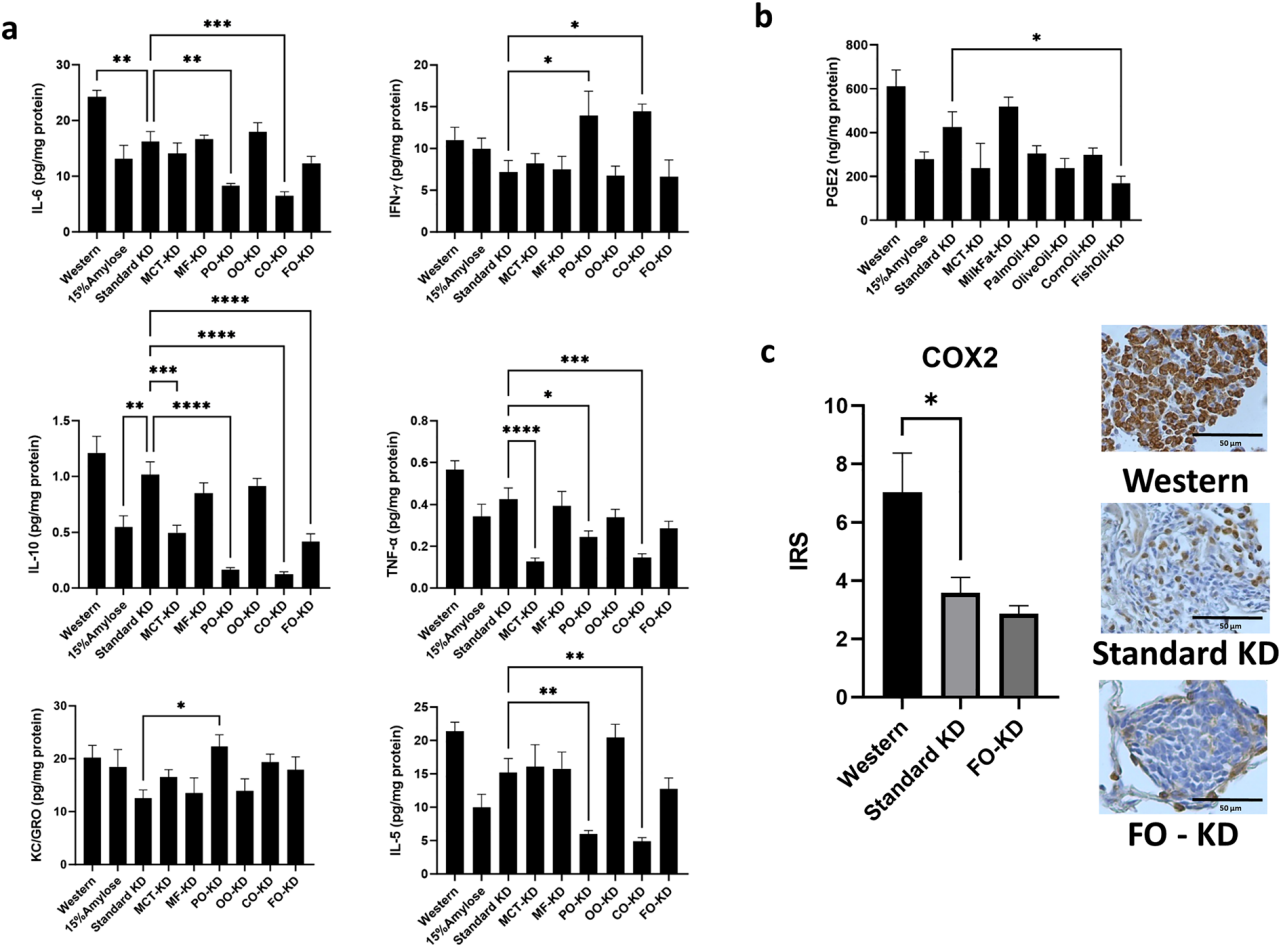
The effect of the various diets on (**a**) proinflammatory cytokines and chemokines, (**b**) PGE_2_ and (**c**) COX2 levels in the lungs. Data shown are from n = 8. Also shown are representative immunohistochemical images. Immunoreactive score (IRS) is a product of their positivity score (attributed 1–4 according to the percent of positive cells), and the intensity score (0–3). *Denotes significance (P < 0.05) compared to mice fed with the standard ketogenic diet.

Since different fats are known to have distinct effects on inflammation^24,25^, we set out to determine if KDs might be modulating lung nodule formation by mediating changes in inflammatory cytokines or chemokines within the lungs. As shown in Fig. 4a, mice fed KDs had significantly (P < 0.05) lower IL-6 levels in their lungs when compared to mice fed a Western diet, suggesting that KDs in general lower inflammation (Fig. 4a). Interestingly, palm oil and corn oil-enriched KDs further lowered IL-6 levels, potentially challenging the long-held notion that palm oil and corn oil are pro-inflammatory^26–29^. On the other hand, the PO-KD and CO-KD resulted in the highest levels of IFNγ and the lowest levels of IL-5 and IL-10 amongst the different KDs. Unexpectedly, even though the FO-KD was the most effective in preventing lung nodule formation, this diet had no unique impact on the pro-inflammatory cytokines/chemokines tested, other than a trend towards high IL-1β levels. Shown in Suppl Fig. 2 are cytokines and chemokines that displayed no significant differences, including IL-1β, IL-2, IL-4, IL-12p70.

Since FO is a rich source of the omega 3 fatty acids, EPA, and DHA, which are known to inhibit the COX2 signaling pathway and thus the levels of the pro-inflammatory lipid mediator, PGE_2_, we also quantified PGE_2_ levels in mouse lungs. Relative to mice fed the standard KD, only the FO-KD significantly (P < 0.05) decreased PGE_2_ levels in mouse lungs (Fig. 4b). It is thus possible this reduction in PGE_2_ may play an important role in the lower tumor formation observed with the FO-KD. To confirm the involvement of the COX2 signaling pathway, we evaluated the expression of cyclooxygenase 2 (COX2) in the lung tumors of the mice on the standard KD and the FO-KD via immunohistochemistry. Interestingly, we found that the standard KD alone significantly (P < 0.05) lowered COX2 expression relative to the Western diet, while the FO-KD had no further significant impact on COX2 expression (Fig. 4c, representative images were taken at 20x magnification). Since the FO-KD significantly lowered PGE_2_ levels relative to the standard KD, the reduction in PGE_2_ may be attributable not to a lower expression of COX2, but to the EPA/DHA outcompeting AA for incorporation into the membrane, which in turn lowers the conversion of AA to PGE_2_ by COX2.

### A ketogenic diet lowers lung fatty acid synthase, and fish oil lowers it further

**Figure 5:**
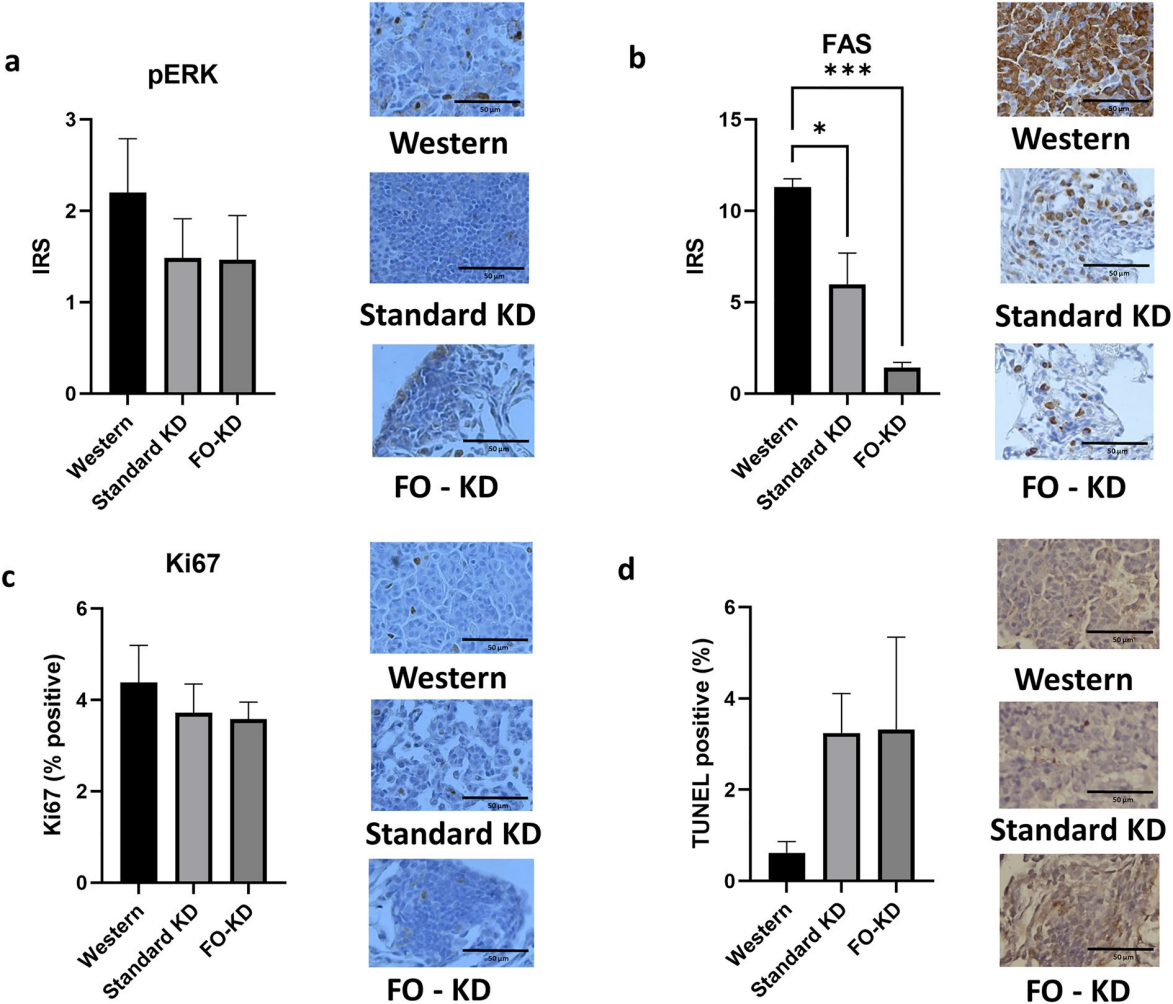
The effect of the Western, standard ketogenic diet and fish oil-enriched ketogenic diet on (**a**) pERK, (**b**) FAS, (**c**) KI67 and (**d**) apoptosis levels in lung tumors. Data shown are from n = 8. Also shown are representative immunohistochemical images. Immunoreactive score (IRS) is a product of their positivity score (attributed 1–4 according to the percent of positive cells), and the intensity score (0–3). *Denotes significance (P < 0.05) compared to mice fed with the standard ketogenic diet. Representative images were taken at 20x magnification.

To determine whether the diets affected signaling pathways within the tumors, we evaluated the expression of pERK. As shown in Fig. 5a, the standard KD and the FO-KD appeared to lower pERK expression relative to the Western diet within the tumors, but this trend did not reach statistical significance.

To assess if the KDs induced a metabolic shift at the tumor level, we measured the expression of FAS in the lung tumors via immunohistochemistry and found that, relative to the mice on the Western diet, the standard KD significantly (P < 0.05) lowered the expression of FAS in the tumor (Fig. 5b). Incorporating FO into the KD further lowered tumor FAS expression, highlighting the potential ability of FO to promote ketogenesis beyond what can be achieved by other fats. This supports the notion that a standard KD and a FO-KD may suppress tumor formation by promoting a metabolic shift that increases fatty acid oxidation as a nutrient source.

We next determined if the reduction in tumor nodules paralleled changes in tumor proliferation, as determined via Ki67, and/or apoptosis, as measured by a TUNEL assay. While there was a trend suggesting the standard KD and the FO-KD reduced Ki67 levels within the tumors of these mice, this decrease was not significant (Fig. 5c). Similarly, there was a trend towards an increased apoptosis within the tumors of mice fed the KDs but the large error bars precluded the differences reaching significance (Fig. 5d). Nevertheless, these trends suggest that the KDs may be lowering tumor formation to some degree by promoting apoptosis and reducing proliferation within these tumors.

### Ketogenic diets do not induce liver damage

**Figure 6:**
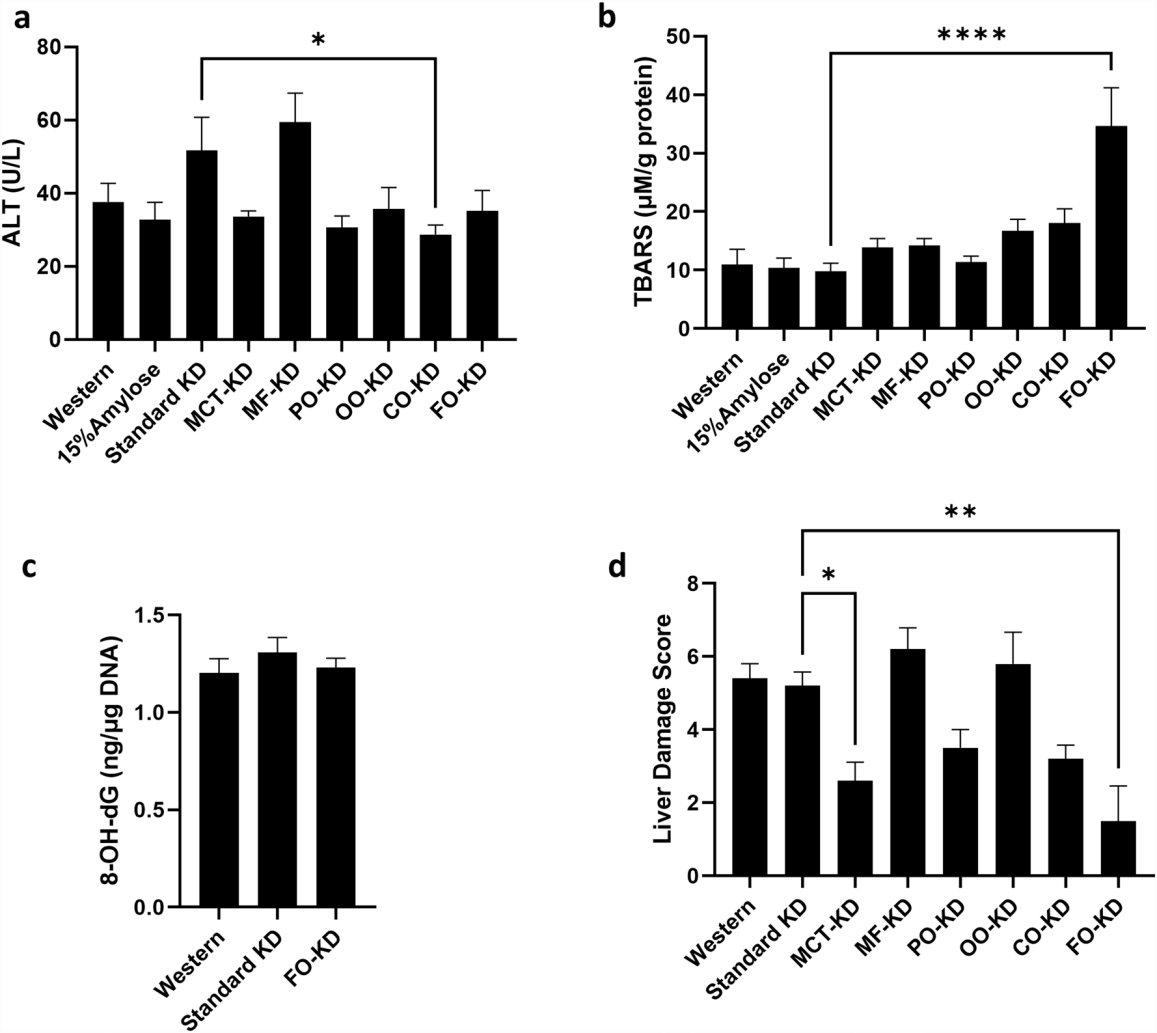
The ketogenic diets do not promote liver toxicity. (**a**) Plasma ALT levels, (**b**) liver TBARS values, (**c**) liver 8-OHdG DNA damage marker and (**d**) liver damage scores by immunohistochemistry (n = 5–9). *Denotes significance (P < 0.05) compared to mice fed with the standard ketogenic diet.

Since consumption of high-fat diets has been associated with the development of non-alcoholic steatohepatitis^30^, we asked if the different KDs may, as an unwanted side effect, induce liver damage over time. Using plasma alanine transaminase (ALT) as a marker of liver injury, blood was taken 1 week before euthanasia and the plasmas from the mice on the different diets evaluated. As shown in Fig. 6a, the ALT levels were not significantly (P < 0.05) higher on the KDs, compared to the Western diet, although there was a trend toward higher ALT levels with the standard KD and the MF-KD (Fig. 6a). Furthermore, when different types of fats were incorporated into the KDs, only corn oil significantly (P < 0.05) affected ALT levels, and did so, surprisingly, by lowering it.

We also evaluated the effect of the diets on the oxidative status of the liver using the TBARS assay. While most KDs did not increase the TBARS value when compared to the standard KD or the Western diet, mice on the FO-KD showed a significant (P < 0.05) increase in TBARS within their livers (Fig. 6b). However, since the TBARS assay is relatively nonspecific for detecting malondialdehyde, the secondary lipid oxidation product measured in the TBARS assay, we performed another assay to measure oxidative stress/damage in the liver. Specifically, we used 8-OH-dG, an indicator of oxidative stress-induced DNA damage, and found that the high TBARS value in the FO-KD did not correspond to increased DNA damage (Fig. 6c). It is therefore possible that the high TBARS value in the livers of mice on the FO-KD may reflect an increased level of fatty acid oxidation rather than an increase in free radical-induced oxidative stress. To further assess the extent of liver damage that could be caused by the KDs, we assessed liver histopathology, based on criteria typically used to establish the presence of non-alcoholic steatohepatitis. As shown in Fig. 6d, mice on various KDs did not increase markers for liver damage when compared to mice fed the Western diet. Interestingly, mice fed the FO-KD had significantly lower liver damage scores than those fed the standard KD. When the individual criteria that make up the liver damage score were evaluated, the FO-KD tended to reduce the scores on every criterion tested, in a non-significant fashion, but significantly lowered liver ballooning degeneration (Suppl Fig. 3). Taken together, considering that these A/J mice consumed their diets for a relatively extended period (22 weeks), we suggest that consumption of KDs did not induce liver damage.

### Ketogenic diets impact the lipid profile

**Figure 7:**
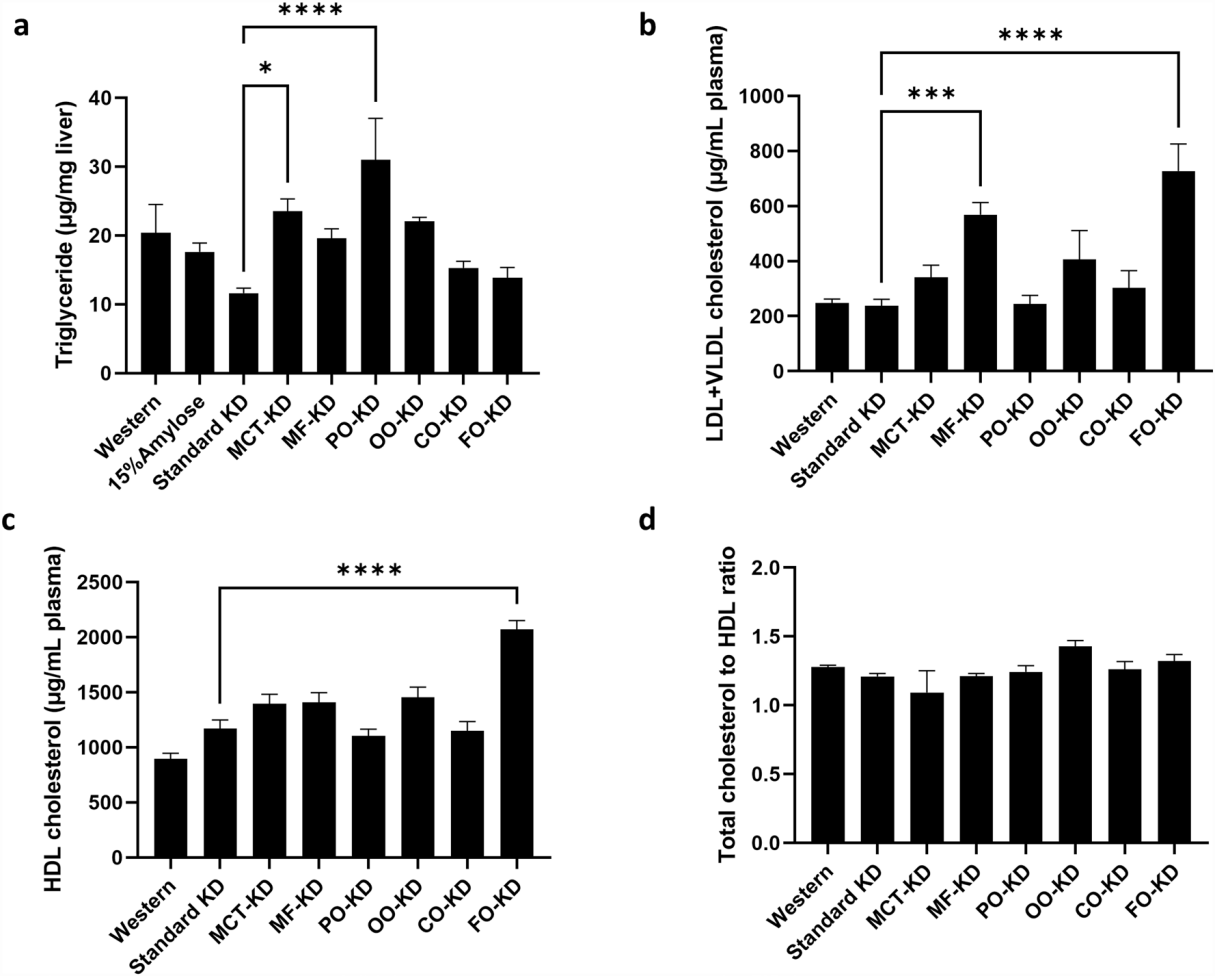
The various diets impact the lipid profile. (**a**) Triglyceride levels. (**b**) LDL + VLDL cholesterol levels. (**c**) HDL cholesterol levels. (**d**) Total cholesterol/HDL (n = 3–8). *Denotes significance (P < 0.05) compared to mice fed with the standard ketogenic diet.

Since the KDs contained high amounts of fat, we asked if this might cause changes to the lipid profiles of the mice. As shown in Fig. 7a, the standard KD tended to decrease the triglyceride level in the liver, compared to the other diets. Incorporation of the MCT oil and palm oil into this KD, however, significantly (P < 0.05) increased triglyceride levels (Fig. 7a).

Looking at cholesterol levels in plasma, the standard KD had no impact on either the LDL + VLDL or the HDL levels when compared to the Western diet (Fig. 7b,c). However, when MF or FO was the dominant type of fat in the KD, we observed a significant (P < 0.05) increase in the levels of LDL + VLDL in the plasma (Fig. 7b). At the same time, we observed a significant (P < 0.05) promotion of HDL levels in the plasma of mice fed the FO-KD (Fig. 7c). When the cholesterol levels were expressed as the total cholesterol/HDL ratio, all of the KDs showed similar ratios (Fig. 7d).

### Fish oil in the ketogenic diet promotes an increase in Akkermansia within the gut microbiome

**Figure 8:**
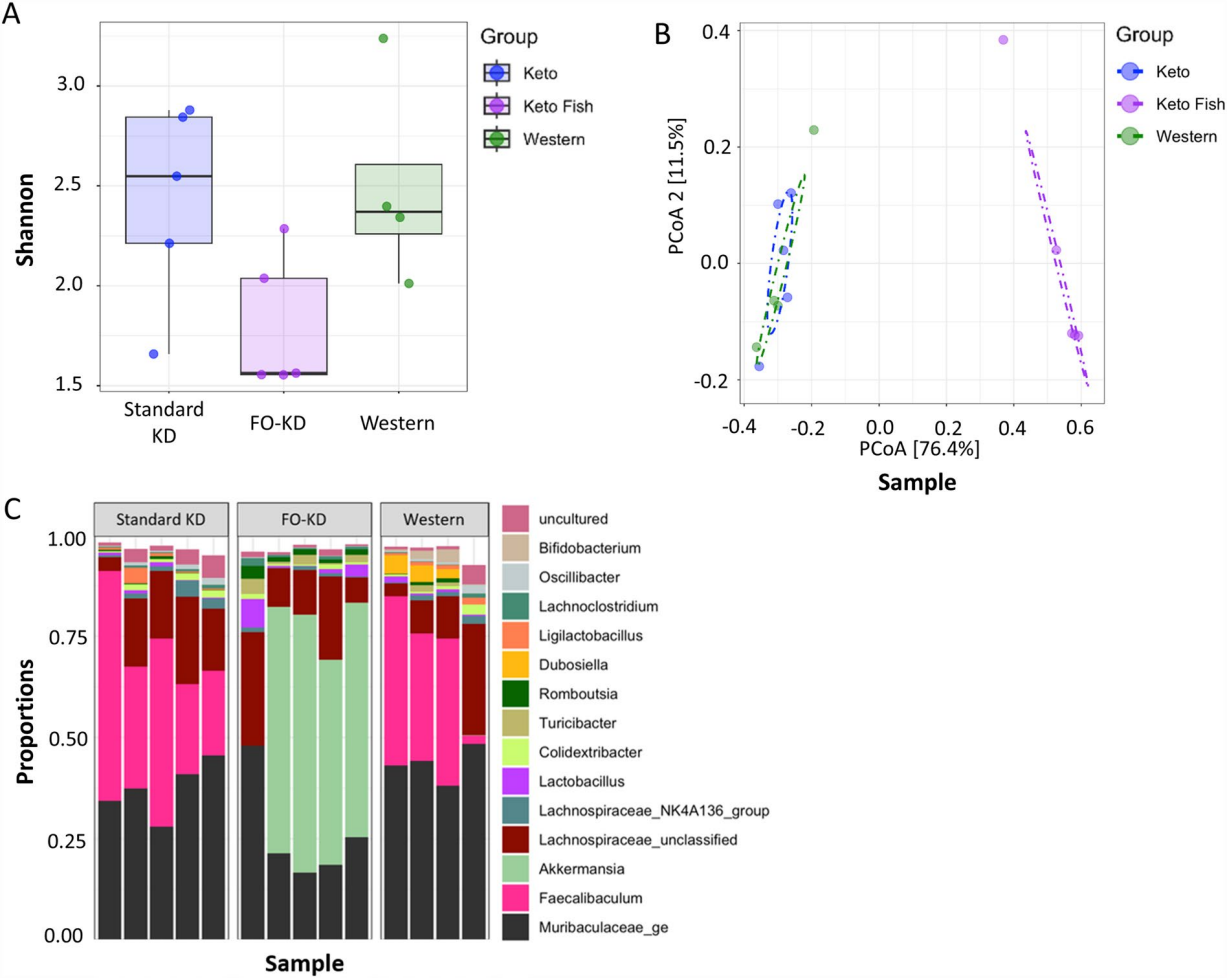
The effect of diets on fecal microbiome composition. While the various diets (**A**) had no impact on the alpha (Shannon) diversity index, (**B**) the FO-KD significantly (P < 0.05) altered fecal microbiome composition visualized using Principal Coordinate Analysis (PCoA) ordination (**C**) and uniquely promotes the growth of the Akkermansia genus in the ketogenic diet containing fish oil (n = 4–5). A dot-dash circle in the PCoA ordination plot represents the center of each cluster^55^.

Lastly, we wanted to look at the effect of the different diets on the gut microbiome since it is well known that changes to the microbiome can dramatically affect inflammatory responses^31,32^. For these studies we compared the 16s sequencing profiles of feces from mice on a Western diet versus the standard KD and the FO-KD. As can be seen in Fig. 8A, no significant differences were found in the Alpha (Shannon) diversity index, suggesting the richness and evenness of the microbial community between groups fed with different diets were comparable. The microbiome composition in the FO-KD diet, however, was found in beta diversity analysis to be significantly (P < 0.05) different from that found in the Western or standard KD diets (Fig. 8B). Adding FO to the KD, led to a dramatic expansion of the genus Akkermansia and a concurrent depletion of the genus Faecalibaculum (Fig. 8C, Suppl Fig. 4). Other minor taxa that were affected by FO addition in the KD diet include the genus Romboutsia, which was significantly (P < 0.05) upregulated, and the genus Ligilactobacilus and Oscillibacter, which were significantly (P < 0.05) downregulated compared to the standard KD diet (Fig. 8C).

## Discussion

In the current study we confirmed our earlier results showing that a 15%Amylose diet significantly lowers NNK-induced lung nodule numbers, compared to a Western diet, in A/J mice^13^, reducing nodule numbers in the current study from an average of 18 ± 3 on the Western diet to 11 ± 4. Moreover, putting mice on a standard KD made up of the same Western-style fats as in the 15%Amylose diet, reduced the average nodule count further to 5 ± 2. Related to this, even though the post-prandial blood glucose levels of the mice on the seven KDs were comparable to that found in mice on the 15%Amylose diet, there was a significant (P < 0.05) increase in plasma β-HB and a significant reduction in liver FAS expression on the KDs. These results suggest a systemic shift in metabolism from glucose utilization to dependence on fats and ketone bodies (KBs) for energy. This promotion of ketogenesis by the KDs could provide an explanation, at least in part, for the superior efficacy of the KDs in reducing lung nodules.

Interestingly, we found that only a KD enriched in FO was more effective than the standard KD in lowering the number of lung nodules. This FO-KD was also the only KD that significantly (P < 0.05) increased plasma β-HB, lowered blood glucose, and lowered the expression of FAS in both the lungs and liver when compared to the standard KD. FO, therefore, appears to be unique in its ability to augment the impact of a KD on mouse metabolism. One possible reason is that of all fatty acids tested, the omega 3 fatty acids, EPA and DHA, increased fatty acid oxidation to a greater extent than other fatty acids. This is consistent with a study carried out by Halvorsen et al.^33^ which showed that an omega-3-enriched diet prominently increased the activity of enzymes involved in mitochondrial (CPT-I and CPT-II) and peroxisomal β-oxidation, when compared to a diets enriched in saturated or mono-unsaturated fats. At the same time, the EPA and DHA in FO could be more potent than other fatty acids in downregulating the sterol regulatory element-binding protein 1c (SREPB1c), a transcription factor that upregulates lipogenic enzymes such as FAS in response to insulin. In support of this hypothesis, the ability of fatty acids to decrease the nuclear content of SREBP-1 in HEK-293 cells was shown to correspond with increasing fatty acid chain length and degree of unsaturation. Tested in primary rat hepatocyte cultures, EPA and DHA, but not oleic acid, prevented insulin-induced SREBP-1c transcription^34^. Compared to safflower oil, which is high in linoleic acid, FO consumption also lowered the mature form of SREBP-1^35^. Related to this, when we compared the fatty acid profiles in the lungs of mice on the different KDs we found they reflected the levels of fatty acids in their chows. This indicates that they are all bioavailable for incorporation into lung tissues and they are not all completely degraded for energy. Specifically for the FO-KD, we observed that, despite a higher EPA level than DHA in the FO-KD diet, DHA was present at more than twice the EPA level in the mouse lungs, suggesting that EPA is likely preferentially oxidized. This is consistent with a report showing greater oxidation of [1-^14^C] EPA than [1-^14^C] DHA in rat liver parenchymal cells, isolated peroxisomes, and purified mitochondria^36^. Whether the effect of FO on reducing lung nodule formation in this study is due more to EPA or to DHA is beyond the scope of this study but is a research question that warrants further investigation.

To determine if the efficacy of the KDs on NNK-induced lung nodule formation was mediated, at least in part, via suppression of chronic inflammation, we evaluated the impact of the various KDs on several pro-inflammatory cytokines and chemokines within the lungs. Interestingly, we found that palm- and corn oil-enriched KDs specifically increased the level of the pro-inflammatory cytokine, IFNγ while reducing the anti-inflammatory cytokine, IL-10, consistent with the expected pro-inflammatory effect of these oils. However, they also reduced both IL-6 and IL-5 levels. As well, the CO-KD led to very low levels of the pro-inflammatory cytokines, IL-1β and TNFα. These unexpected results may explain, in part, why CO-KD and PO-KD did not increase NNK-induced lung nodules, compared to the other KDs. Of note, none of the diets affected the lung levels of IL-12 or IL-2 and, more importantly, the FO-KD did not distinguish itself in any way from the other KDs, other than maybe a trend towards increasing IL-1β levels within the lungs. Thus, the superiority of the FO-KD in lowering NNK-induced lung nodules may not be mediated by effects on cytokines and chemokines. The caveat, of course, is that we did not test an exhaustive list of cytokines and chemokines.

To gain further insight into the possible mechanisms responsible for the efficacy of KDs and especially the FO-KD in lowering NNK-induced lung nodules, we examined the effects of the different diets on PGE_2_ and COX2 levels within the lungs. As shown in Fig. 4b and c, the standard KD resulted in a significant reduction in lung COX2 and there was a trend towards a further lowering on the FO-KD. This result is reflected to some extent in the PGE_2_ levels. Specifically, the FO-KD significantly lowered PGE_2_ levels within the lungs compared to the other KDs, suggesting that the efficacy of FO in further lowering lung nodule numbers could be mediated by its unique ability to further suppress PGE_2_. This reduction in PGE_2_ is somewhat expected since the EPA and DHA in the FO are known to compete with AA for incorporation into the cell membrane and, in doing so, prevents COX2 conversion of AA to PGE_2_. This is consistent with our finding that the FO-KD increased the EPA and DHA content in the lungs while, at the same time, reducing AA levels (Fig. 3).

Despite the apparent impact of the diets on the COX2 signaling pathway, we previously reported that celecoxib, a COX-2 inhibitor that reduces PGE_2_ synthesis, was not effective in lowering NNK-induced lung tumor formation^13^. We, therefore, suggest that although suppression of PGE_2_ can contribute to suppression of lung tumor formation, the primary mechanism by which the FO-KD prevents nodule formation is likely metabolically driven. This is highlighted by the observation that the lung tumors of the KD-fed mice had significantly lower FAS, which was further significantly decreased by the FO-KD (Fig. 5b), coincident with the reduction in lung nodule numbers in these mice. The KDs, therefore, are likely causing a metabolic switch within the lungs that favors fatty acid oxidation and reduces reliance on glucose utilization, and the incorporation of FO into the KD enhances this shift.

One of the concerns related to the long-term consumption of KDs is that they typically have high levels of saturated fat and this has been associated with an increase in LDL-cholesterol^16,37^. LDL-cholesterol, in turn, is causally related to an increased risk of cardiovascular disease (CVD)^38^. Saturated fats have also been reported to promote inflammation, which, if consumed long-term could promote chronic low-grade inflammation, and this is strongly linked with the development of cancer^39–41^.

Thus, we wanted to determine if the consumption of these KDs adversely impacted the lipid profile or the health of the liver. To do this we characterized the effect of the diets on plasma triglyceride (TG) and cholesterol levels, as well as evaluated several indicators of liver health, via plasma markers and histopathology of the liver. Based on plasma ALT levels, liver dOHdG levels, and histopathology scores, the standard KD did not appear to induce liver damage. In addition, the liver damage score for mice on the MCT-KD and FO-KD was even lower than mice on the standard KD. This is consistent with previous reports suggesting the usefulness of MCT oil and omega-3 fatty acids in preventing non-alcoholic steatohepatitis in rodents^42^.

While not significant, the standard KD tended to lower triglyceride levels when compared to mice on our Western or 15%Amylose chow. This is consistent with the idea that increased CHO raises TG synthesis^43^. Herein, we also demonstrate that even though the standard KD had more saturated fat than the Western diet, there was no increase in the VLDL + LDL cholesterol. Of the different types of KDs we tested, only the MF-KD and FO-KD significantly raised the VLDL + LDL cholesterol level. While MF-KD contains higher levels of saturated fat than the standard KD, the FO-KD does not, and we suggest that the increase in VLDL + LDL cholesterol on these two diets may be due to increased dietary cholesterol that is inherently higher in animal-based fats, rather than its saturated fat content (Suppl Table 2). Despite the increase in LDL-cholesterol, we also observed a significant increase in HDL levels, especially with the FO-KD. When expressed as a ratio of total cholesterol to HDL, a metric that appears to be a more powerful coronary risk predictor than LDL-cholesterol alone^44^, none of the KDs increased this ratio. In addition, while it is important to note that while LDL-cholesterol is indeed related to atherosclerotic cardiovascular disease (ASCVD), the number of apoB particles in plasma, rather than the LDL-cholesterol, appears to be a more accurate marker of cardiovascular risk^45^. In addition, the apoB/apoA-I (the principal apolipoprotein in LDL and HDL, respectively) ratio has been found to be stronger than the total cholesterol/HDL cholesterol in predicting atherogenic risk^44,46^. Further research on the impact of the KD on these end point measures is likely warranted to determine if the increase in LDL + VLDL observed with the MF-KD and FO-KD increases cardiometabolic risk.

Our finding that the FO-KD increases the proportion of fecal Akkermansia is consistent with recent findings suggesting that the Gram-negative anaerobic bacterium, *Akkermansia muciniphila*, is increased in both humans^47^ and mice^48,49^ in response to omega 3 fatty acid supplementation. This genus of gram-negative bacteria has been purported to have many health benefits^50,51^. For example, *A. muciniphila* has previously been reported to increase anti-tumor immunosurveillance, likely via stimulation of pattern recognition receptors (PRRs) such as Toll-like receptor 2 (TLR2) and TLR4. In addition, *A. muciniphila* has been shown to enhance the production of the purine metabolite, inosine, which promotes Th1 immune cell differentiation, thus enhancing the efficacy of immunotherapies for multiple cancers in murine models^50^.

Our observation that the FO-KD diet depleted Faecalibaculum is also intriguing since their absolute abundance has been positively associated with obesity and hyperglycemia^52^. This may be related to a recent report showing that sugar promotes the outgrowth of the one species known to belong to the Faecalibaculum genus, *Faecalibaculum rodentium*. There is evidence this species eliminates commensal Th17 cells to increase the risk of metabolic disease^53^. On the other hand, *Faecalibaculum rodentium* has also been recently identified to have an anti-tumor activity in murine models of colorectal cancer, likely by producing short chain fatty acids that inhibit colon cancer proliferation^54^. The exact mechanism by which the FO-containing KD may promote the Akkermansia while depleting Faecalibaculum and whether this directly or indirectly affects lung tumor formation in our model system, however, needs to be established in future research.

To conclude, while KDs are more effective than a 15% amylose CHO diet in reducing NNK-induced lung cancer in A/J mice, the efficacy of the KD is magnified when FO is incorporated into the diet. Although this results in an increase in VLDL + LDL levels, the effect of these KDs on cardiovascular events remains to be determined in future studies.

### Supplementary Information

Supplementary Information.

## Data Availability

All data relevant to the study are included in the article or uploaded as supplementary information.
