# Supplementary material for: A ketogenic diet rich in fish oil is superior to other fats in preventing NNK-induced lung cancer in A/J mice
Source: Sci Rep. 2024 Mar 7;14:5610. doi: 10.1038/s41598-024-55167-6 (PMC10920871; doi:10.1038/s41598-024-55167-6)
Supplement: Supplementary file 1 — Supplementary Information. [file 41598_2024_55167_MOESM1_ESM.pdf]

A Ketogenic Diet Rich in Fish Oil is Superior to Other Fats in Preventing NNK-Induced Lung Cancer in A/J Mice

Ingrid Elisia[1], Michelle Yeung[1], Sara Kowalski[1], Taras Shyp[2], Jason Tee[1], Serena Hollman[1], Amy Wong[1], Janette King[3], Roger Dyer[3], Poul H. Sorensen[2], Gerald Krystal[1*]

[1]The Terry Fox Laboratory, BC Cancer,

Vancouver, British Columbia, Canada

[2] Department of Molecular Oncology,

BC Cancer, Vancouver, British Columbia V5Z 1L3, Canada.

[3]Analytical Core for Metabolomics and Nutrition,

BC Children's Hospital Research Institute, Vancouver, BC, Canada.

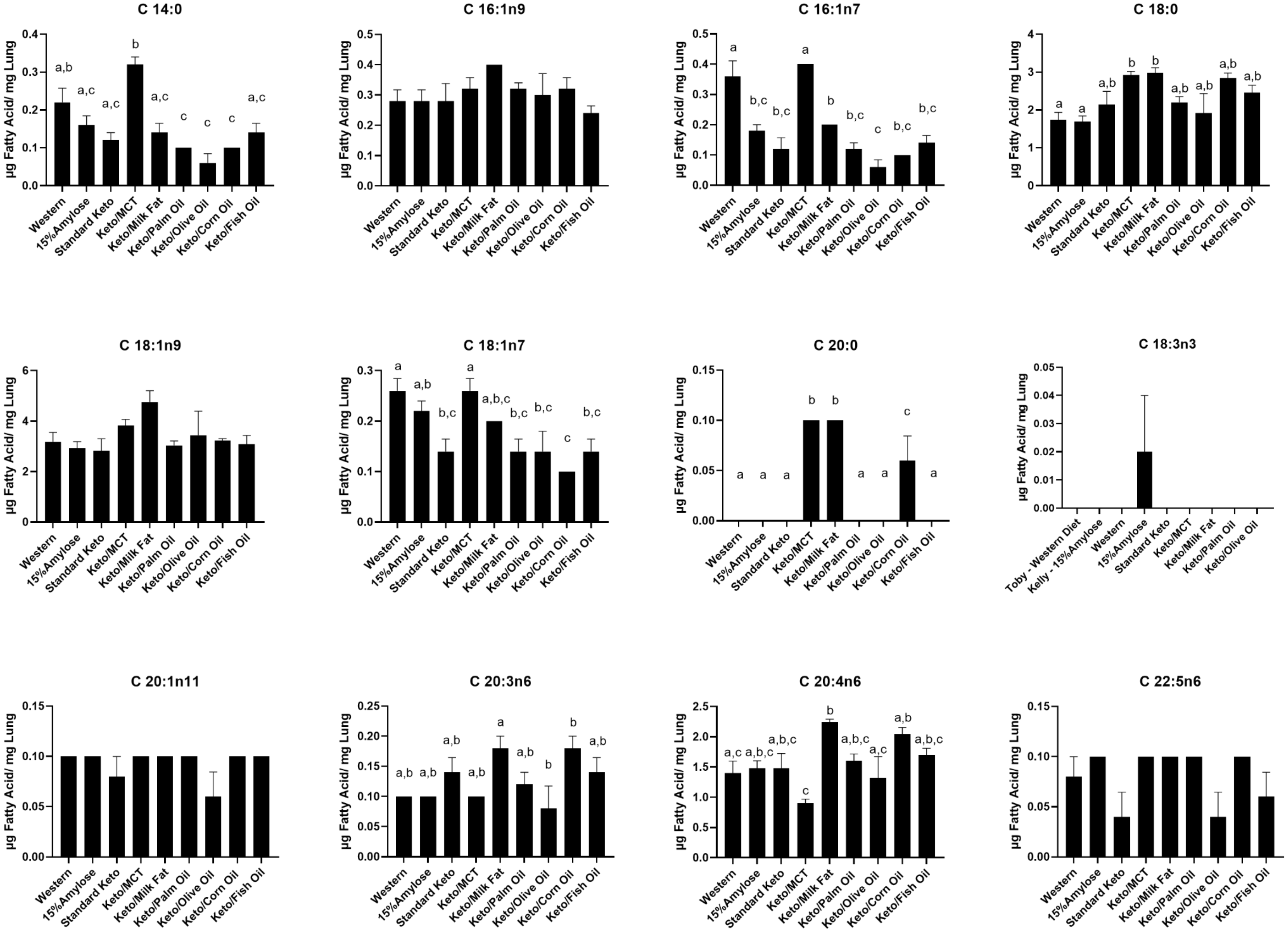

Supplemental Figure 1. The effect of the diets on the lung fatty acid profile.

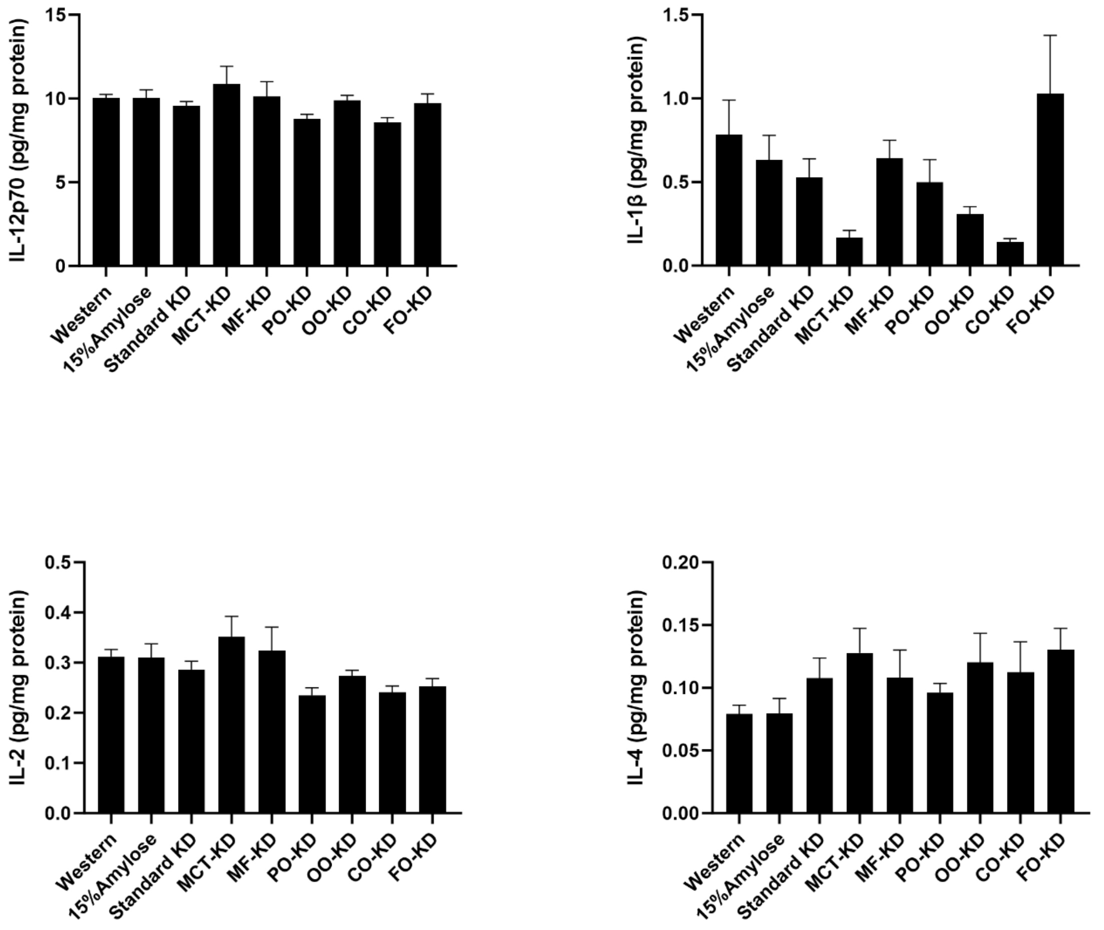

Supplemental Figure 2. The diets had no impact on IL-12p70, IL-1β, IL-2, and IL-4 (n = 8).

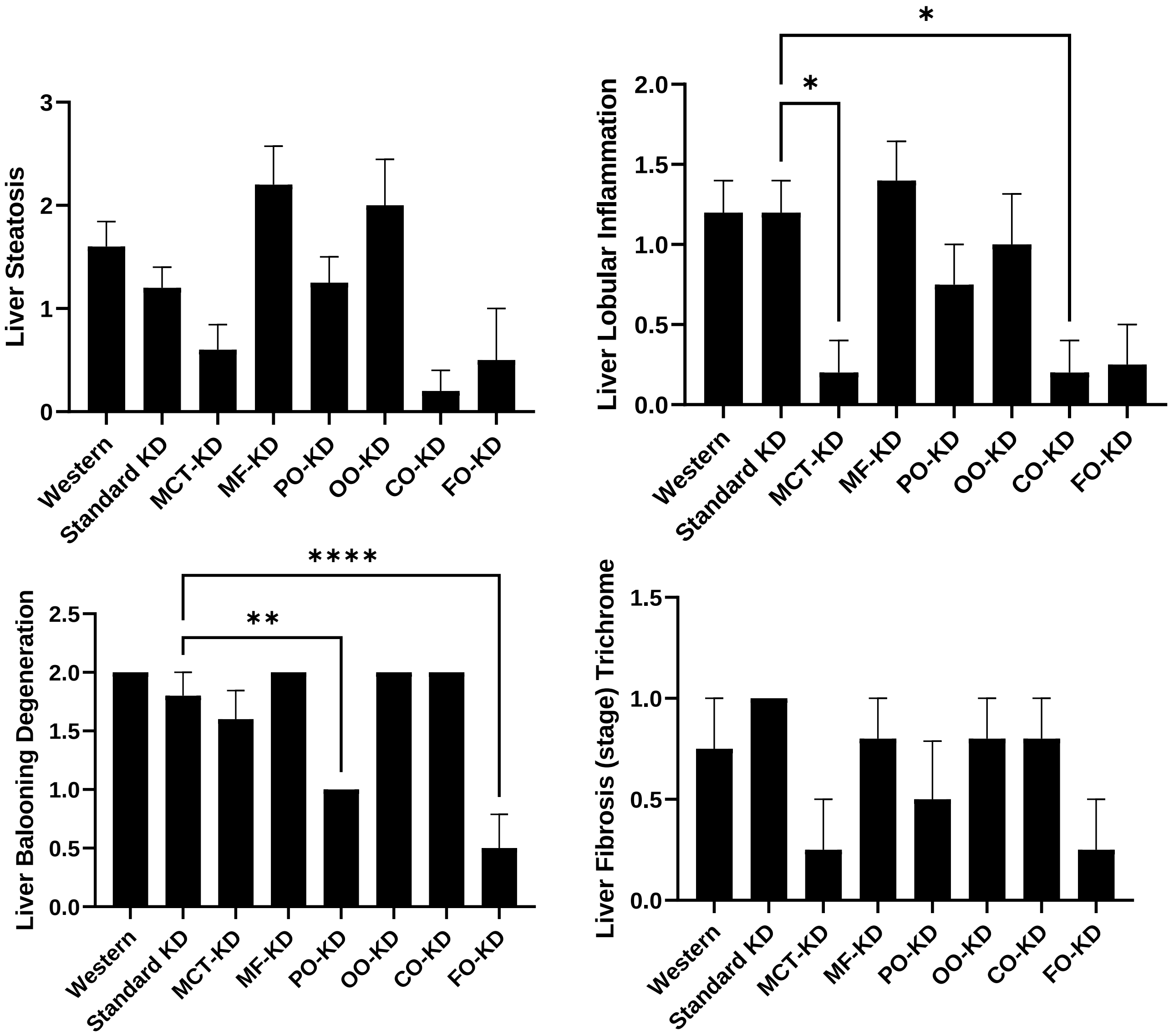

Supplemental Figure 3. The effect of the diets on liver steatosis, lobular inflammation, ballooning degeneration and fibrosis.

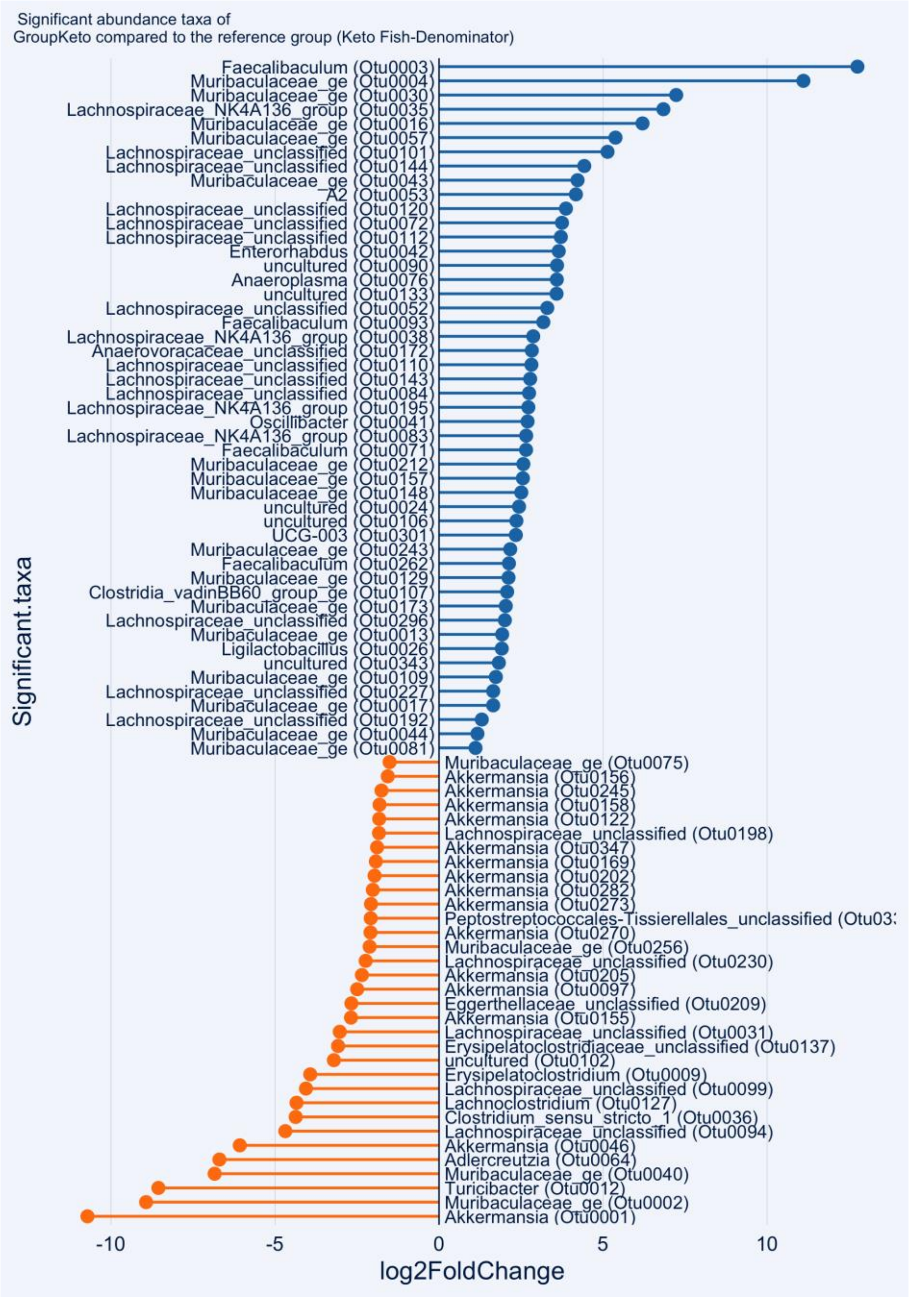

Supplemental Figure 4. Differentially abundant taxa between the standard Keto and FO-KD diets.

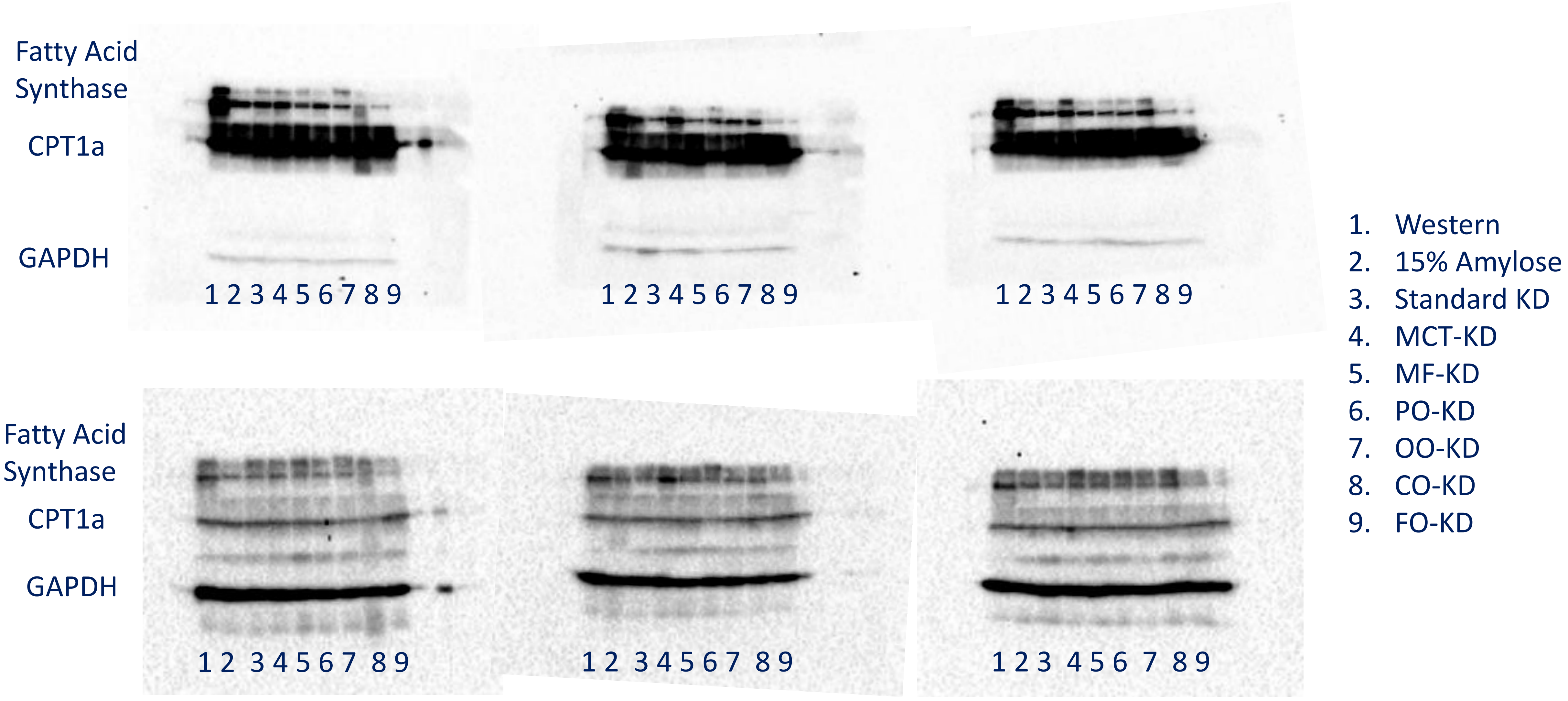

Supplemental Figure 5. Representative uncropped blot probing for liver FAS and CPT1a at different exposure times. GAPDH was used as a loading control. The top blot was used to evaluate the relative expression of fatty acid synthase levels, and the bottom blot was used to evaluate the relative expression of CPT1a and GAPDH levels.

Supplemental Table 1. Comparison of fats and oils used in diets expressed as g/Kg of total fatty acid

	Western	15% Amylose	Standard Ketogenic	Ketogenic MCT Oil	Ketogenic Palm Oil	Ketogenic Corn Oil	Ketogenic Milk Fat	Ketogenic Olive Oil	Ketogenic Fish Oil
**Cocoa butter**			187	187	187	187	187	187	187
**Soybean oil**	31.4	42.1	86.1	42.1	42.1	42.1	42.1	42.1	42.1
**Anhydrous milk fat**	36.3	48.6	99.6	48.6	48.6	48.6	280.2	48.6	48.6
**Olive oil**	28	37.5	76.8	37.5	37.5	37.5	37.5	37.5	37.5
**Lard**	28	37.5	76.8	37.5	37.5	37.5	37.5	37.5	37.5
**Beef tallow**	24.8	33.2	68	33.2	33.2	33.2	33.2	33.2	33.2
**Corn oil**	16.5	22.1	45.3	22.1	22.1	253.6	22.1	22.1	22.1
**Fish oil**									231.5
**Palm oil**					231.5				
**Olive oil**								231.5	
**MCT (C:8)**				231.5					

Supplemental Table 2. Comparison of fatty acid composition used in diets expressed as g/kg of total fatty acid

	Western	15% Amylose	Standard Ketogenic	Ketogenic MCT Oil	Ketogenic Palm Oil	Ketogenic Corn Oil	Ketogenic Milk Fat	Ketogenic Olive Oil	Ketogenic Fish Oil
**4:0 Butyric**	0.7	1.0	2.0	1.0	1.0	1.0	5.7	1.0	1.0
**6:0 Caproic**	0.5	0.7	1.5	0.7	0.7	0.7	4.3	0.7	0.7
**8:0 Caprylic**	0.4	0.5	1.0	237.7	0.5	0.5	2.9	0.5	0.5
**10:0 Capric**	0.9	1.2	2.5	1.2	1.2	1.2	7.1	1.2	1.2
**12:0 Lauric**	1.1	1.5	3.0	1.5	1.5	1.5	8.6	1.5	1.5
**14:0 Myristic**	5	6.7	13.9	6.7	9.1	6.7	32.8	6.7	28.1
**16:0 Palmitic**	31.7	42.5	136.7	91.1	197.8	119.6	159.9	119.6	129.0
**16:1 Palmitoleic**	2.1	2.9	5.9	2.9	2.9	2.9	6.4	5.2	31.3
**18:0 Stearic**	15.2	20.4	107.8	85.9	96.5	90.6	116.7	93.0	93.0
**18:1 Oleic**	63.1	84.6	238.9	148.1	240.6	214.5	212.2	318.9	173.0
**18:2 Linoleic, cis**	35.8	48.0	105.1	53.6	76.1	188.8	63.1	74.9	58.3
**18:3 Linolenic**	3.4	4.6	9.5	4.6	4.6	7.0	5.8	7.0	8.1
**18:4 Stearidonic**									7.1
**20:4 Arachidonic**									3.6
**20:5 EPA**									32
**22:5 DPA**									2.4
**22:6 DHA**									24.9
**Saturated % of fat**	33.3	33.1	41.8	66.1	47.9	34.6	52.5	34.9	39.7
**MUFA % of fat**	39.2	39.0	37.9	23.4	37.7	33.6	34.0	50.1	31.6
**PUFA % of fat**	23.5	23.3	17.7	9.0	12.5	30.2	10.4	12.6	21.1
**n-6:n-3 ratio**	10.5	10.5	11.1	11.7	16.6	27.1	10.9	10.8	0.8
**Cholesterol mg/kg diet**	584.7	675.6	845.1	634.4	634.4	634.4	1241.5	634.4	1870.0
